# The Relationship between Individual-Level and Context-Level Factors and Social Distancing from Patients with Depression in Taiwan: A Multilevel Analysis of National Surveys

**DOI:** 10.3390/ijerph17197109

**Published:** 2020-09-28

**Authors:** Chi-Hsuan Tsai, Yu-Chen Kao, Yin-Ju Lien

**Affiliations:** 1 Department of Health Promotion and Health Education, National Taiwan Normal University, 162, Heping East Road Section 1, Taipei 106, Taiwan; archy.cht@gmail.com; 2 Department of Psychiatry, Tri-Service General Hospital Songshan Branch, National Defense Medical Center, Chenggong Rd., Neihu District, Taipei 114, Taiwan; wheelis001@gmail.com

**Keywords:** public stigma, depression, psychiatric rehabilitation services, urbanization degree, multilevel analysis

## Abstract

*Background:* Research on social distancing from patients with depression has primarily focused on individual-level factors rather than context-level factors. This study aimed to investigate the relationship between individual-level and context-level factors and social distancing from depressive patients. *Methods:* Sample data were collected via computer-assisted telephone interviews with 800 Taiwanese adults aged 20 to 65 years in 2016. All effects were tested using multilevel analysis. *Results:* With regard to individual-level variables, male sex, older age, people with more perceived dangerousness and those with more emotional reaction of fear were associated with greater social distancing from depressive patients. After controlling for individual-level variables, a positive association was found between the degree of urbanization and social distancing. We also found the interaction between the density of psychiatric rehabilitation services and perceived dangerousness to be associated with social distance. This finding revealed that persons with more perceived dangerousness and living in a region with higher density of psychiatric rehabilitation services were associated with greater social distance. *Conclusions:* We found that social distancing from depressive patients is not only determined by individual-level factors but influenced by the surroundings. This study provides useful directions for the implementation of optimal anti-stigma interventions for patients with depression.

## 1. Introduction

Depression is a significant public health issue due to its high prevalence [1] and high economic burden [2]. In its most severe form, depression can lead to suicide [3]. Effective and evidence-based treatments for depression such as psychotherapy and pharmacological treatments are available [4]. However, many people do not receive professional care for their symptoms. It is estimated that more than half of those who suffer from depression worldwide are unwilling to seek appropriate medical help [5]. The stigma of mental illness has often been considered a potential cause for reluctance in seeking help [6]. There is evidence that depression can run in families, suggesting that genetic factors contribute to the risk of developing depression and exacerbating the stigma of depression [7,8]. In addition, depression is not episodic, but chronic and admixed with anxiety and becomes rather indistinguishable from personality [9]. These characteristics are more frequently associated with stigmatizing attitudes [9]. Hyposensitivity or hypersensitivity may be “trait” markers of individuals with major affective disorders and interventions should refer to the individual unique sensory profiles and their behavioral and functional impact in the context of real life [10]. The ultimate goal of reducing stigma towards mental illness is to change behavior (reduce discrimination) towards individuals with mental illness [11]. In previous studies, the desire for social distance has been used to measure the level of discrimination against people with mental illnesses [12,13]. Recent research in Taiwan has shown that people maintain a certain social distance from people with depression. For instance, 37% of the people are unwilling to work closely with depressive patients [14].

Previous studies have suggested that sociodemographic characteristics, including sex, age and educational attainment, were associated with social distance [15]. Females were found to be more empathetic towards persons with mental illness than males [16]. Most studies have shown that men maintain greater social distance from people with depression than women [17]. Concerning age, older people have a higher tendency to stay away from people with depression than younger people [18,19]. Educational attainment reflects socioeconomic status or social inequality [20]. A systematic literature review revealed inconsistent results between factors of educational attainment and public attitudes towards people with mental illness, with some results showing that people with a high educational level do not tend to distance themselves from patients with mental illness, while others have shown no relationship between educational attainment and social distance [21].

Other than sociodemographic characteristics, different stigma components (e.g., stereotypes and prejudice) may also affect social distance [22,23]. Stereotypes include beliefs one holds about what it means to have a mental illness, while prejudices include agreement with the stereotypes, which results in an emotional reaction of some kind [15,24]. Many studies have found that people with more perceived dangerousness towards people with mental illness, increasing the level of social distancing from them [22,25,26,27]. When people’s emotional reactions of fear increase, the acceptance of depressed patients decreases, in turn increasing the social distance from them [23,28].

Substantial literature exploring the relationship between these variables is mostly conducted in Western countries [22,28]. To date, studies examining public attitudes towards people with mental illness are rare in the Chinese community [29]. However, the stigma of depression is severe in the Chinese community, with a negative attitude towards people with mental illness [30]. Chinese Confucian culture places great emphasis on the concepts of harmony [31] and collectivism [32], which may affect individuals’ behaviors and attitudes towards mental health issues [33]. Likewise, the importance of “maintaining face” (dignity, reputation, status and public image) is common in the Chinese community [33]. Chinese people are concerned that socializing with people with mental illness would result in losing face even with limited interaction or association with people with mental illness [34].

In addition to individual-level variables, differences across regional structures may lead to different forms of stigmatization towards mental illness in diverse regions [35]. However, most of the surveys on the stigma of mental illness focus on individual-level data [36], without considering the differences at the context level, thus presenting a limited final result [37]. The following context variables are the common factors that influence mental illness stigma: the distribution of psychiatric rehabilitation services [34] and the level of economic and environmental development [38]. The establishment of psychiatric rehabilitation services in the community has frequently been greeted with protests from the residents in Taiwan [39] as well as in other countries [40]. It has been found that psychiatric rehabilitation services have been categorized as one of the “not in my back yard” (NIMBY) facilities. Therefore, people living nearby tend to avoid these facilities in their area [41]. The effect of ostracization from community residents reflects the fact that they still have concerns about mental illness [42]. A qualitative study postulated that the NIMBY phenomenon may be related to the stigma of mental illness [43]. However, very few evidence-based studies have directly clarified the relationship between these two factors to date. Furthermore, the degree of urbanization can also reflect the level of economic and environmental development in the community [44]. Previous studies have found that the correlation between urbanization degree and stigma is inconsistent. Some studies indicated that residents living in rural areas have a greater stigma towards patients with mental illness than urban residents [45,46], while some studies pointed out that urban residents tend to keep social distancing from patients with mental illness [47,48].

Previous studies on mental illness stigma have mainly been conducted in Western countries and have focused on individual-level factors rather than context-level factors. The effect of cross-level interaction on mental illness stigma remains unknown. When testing whether the contextual effects of mental health-related attitudes exist, a multilevel analysis should be conducted to control the variation across different regions [37]. This study investigates the relationship between individual-level and context-level factors and social distancing from patients with depression among the general public using a multilevel analysis. We assume that the social distance of depression is related to personal sociodemographic characteristics, stereotype, prejudice and the contextual factors of residential area, such as psychiatric rehabilitation services density and urbanization degree. Furthermore, there are interaction effects between individual-level and context-level variables on social distance.

## 2. Materials and Methods

### 2.1. Sample Design

Research data were generated from a nationwide survey on public beliefs and attitudes towards schizophrenia and depression [49]. Cross-sectional data were obtained through random-digit-dialing computer-assisted telephone interviews [50] conducted over 1 month (October–November 2016). Personal fully structured interviews were administered by well-trained employees of a large Taiwanese opinion polling firm, all of whom were fluent in a local language (i.e., Mandarin or Taiwanese). The estimated population of Taiwan in October 2016 was 23,526,295. The target population for this study was 16,142,984 (adults aged 20–65 years), representing 68.6% of the aforementioned Taiwanese population.

The sample consisted of persons aged 20 to 65 years living in private households with conventional telephone connection in all administrative districts in Taiwan (i.e., 26 cities and counties). The sample was randomly drawn from all registered private telephone numbers. Operationally, the respondents were selected using a multistage stratified random sampling technique, according to sex, age and place of residence. Of 2,145 individuals, 545 individuals refused to respond, yielding an effective completion rate of 74.6%. The final sample size was 1600. Randomly drawn subsamples were presented with either the vignette entailing schizophrenia (*n* = 800) or depression (*n* = 800). There were no significant differences in sex, age and place of residence between the Taiwanese population and the survey sample [49]. This study analyzed 800 questionnaires based on public beliefs and attitudes towards patients with depression. Before data collection, the study was approved by the Tri-Service General Hospital (ID: 1-105-05-085) responsible for the protection of human participants.

### 2.2. Measures

The interview content was based on a vignette of a person with depression according to the criteria described in the Diagnostic and Statistical Manual of Mental Disorders, Fifth Edition [51], without the use of clinical terminology. Respondents read a vignette of the same sex as their own. The vignette of the male was referred to as “Daxiong” and the one of the female, as “Mei”. Before use, the vignettes was submitted to four psychiatrists and psychologists for blind diagnostic allocation. All experts arrived at consistent diagnoses for the vignettes (see Appendix A for details).

#### 2.2.1. Dependent Variable

The social distance scale [52] measured the willingness (1 = definitely willing, 5 = definitely unwilling) of respondents to (1) rent out a room to the person in the vignette, (2) accept the person as a coworker, (3) accept the person as a neighbor, (4) hire the person for taking care of their children, (5) accept the person as an in-law, (6) introduce the person to their friend and (7) recommend the person if a friend was looking for an employee, with high scores indicating a preference for increased social distance. It showed good internal validity in past research, with a Cronbach’s *α* of 0.75 [53,54]. In the current sample, this tool demonstrated strong internal consistency (Cronbach’s *α* = 0.78).

#### 2.2.2. Individual-Level Variables

Sociodemographic characteristics included sex, age and educational attainment. Sex was divided into “male” or “female”; the age was divided into groups of “20–29 years”, “30–39 years”, “40–49 years”, “50–59 years” and “60–65 years” while educational attainment was categorized as “elementary school or below”, “junior high school”, “high school or vocational school” and “university or higher”.

Perceived dangerousness was measured with the subscale of the personal attributes scale [55,56]. In this study, two of the items were used to measure perceived dangerousness (i.e., “the person in vignette is dangerous”, “the person in the vignette is aggressive or violent”). A 5-point Likert scale (1 = strongly disagree, 5 = strongly agree) was provided for responses and a composite (with higher values indicating higher perceived dangerousness) was derived by totaling the sum of all items. The correlation coefficient between the two items was 0.53, showing moderate-to-strong correlation [57].

The emotional reaction of fear was assessed using the emotional reaction to mental illness scale [58], which has acceptable internal validity in past research, with a Cronbach’s alpha value of 0.78 for fear. In this study, one of the questions was used to measure the emotional reaction of fear. The item was that “The vignette person scares me” The respondents’ emotional reaction of fear to the individuals described in the vignette was assessed on a 5-point Likert scale (1 = strongly disagree, 5 = strongly agree). The higher the score, people with more emotional reactions of fear towards patients with depression.

#### 2.2.3. Context-Level Variables

The density of psychiatric rehabilitation services was based on public information from the Department of Statistics in the Ministry of Health and Welfare [59]. The number of psychiatric rehabilitation services in 18 counties in 2016 was compiled taking into account the population of each county in 2016 to obtain the density of psychiatric rehabilitation services (the number of mental rehabilitation institutions per 100,000 people). The mental rehabilitation institutions mentioned above include community rehabilitation centers, halfway houses and psychiatric nursing homes.

Degree of urbanization refers to the proportion of people living in an urban area. Previous research has pointed out that in Taiwan, urban planning areas are categorized as urban areas, while others are regarded as rural areas. Therefore, in this study, urbanization degree was estimated by dividing the number of residents living in urban counties in 2016 by the total population of the counties in 2016. The data were obtained from the Department of Statistics, Ministry of the Interior [60].

### 2.3. Statistical Analysis

The analysis was conducted using SPSS software version 23.0 [61]. To determine which individual-level variables would be included in the subsequent multilevel analysis, we carried out a bivariate correlation analysis with individual-level data to examine the statistical relationship between each explanatory variable and social distance. For the analysis, three types of correlation analyses were used depending on the types of variables being studied. We examined the correlation between perceived dangerousness, emotional reaction of fear and social distance using Pearson’s correlation coefficient. Spearman’s rank correlation analysis was performed to define the correlation between age, educational attainment and social distance. We also calculated the point bi-serial correlation to test the association between sex and social distance.

In the second phase, a multilevel analysis was used to test whether the individual-level and context-level characteristics were associated with social distancing from patients with depression. Intraclass correlation coefficient (ICC) was calculated to ascertain whether or not a multilevel model is necessary given the study data. In standard regression modeling, an assumption is made that the observations are independent. However, this assumption was violated in the current study because social distancing from patients with depression from the same county had the same values on objective context characteristics. A multilevel approach was an ideal model that could be used to evaluate two-level nested data, as it does not require the variables to be independent.

Multilevel regression models were tested for the influences of individual-level and context-level variables on the social distancing from depression patients among the general public. We further examined whether the interaction between individual-level and contextual-level variables had an effect on the social distancing from depression. The linear mixed-effects models (MIXED) procedure of SPSS was used for these analyses. A *p*-value of less than 0.05 was determined for statistical significance.

## 3. Results

Table 1 presents the descriptive statistics of the participant characteristics. Of the 800 respondents, 50% were male. The majority (24.00%) were aged 30–39 years; the level of education of the majority (61.25%) was college or higher, followed by high school (30.25%). Perceived dangerousness (skewness = 0.52; kurtosis = −0.62), emotional reaction of fear (skewness = 0.55; kurtosis = −1.09) and social distance (skewness = 0.56; kurtosis = 0.28) were considered to be normally distributed when skewness values were between −1 and 1 and kurtosis values were between −2 and 2 [62,63].

Table 2 illustrates the correlations between social distance and all individual-level variables. Men desired more social distancing from patients with depression than women (*r* = −0.07). There were positive correlations between social distance and age (*r* = 0.23), perceived dangerousness (*r* = 0.22) and emotional reaction of fear (*r* = 0.20). There was no relation between the level of education and social distance. Therefore, educational attainment was not included in subsequent analyses.

Table 3 shows the results of multilevel analysis, which was performed to examine the association between individual and context factors. First, the results of the null model (Model 1) indicated a small, but significant association between people in different regions and social distance (intraclass correlation [ICC] = 1.19%). Thus, we proceeded with multilevel modeling to account for this variance. The density of psychiatric rehabilitation services and the degree of urbanization were enrolled as context-level variables in this study. The second model (Model 2) included the individual-level variables which are based on the bivariate correlation results. This model shows that all individual-level variables are significantly associated with social distancing from patients with depression. Compared to female participants, male participants had high social distance. Age was positively associated with social distance. People with high perceived dangerousness and those with an emotional reaction of fear were associated with high social distancing from patients with depression. In Model 3, in which all individual-level variables were included, the association between psychiatric rehabilitation services density and social distance was statistically insignificant. The degree of urbanization was significantly associated with social distance. That is, the higher the urbanization degree in counties and cities, the greater the social distancing from patients with depression. In the final model (Model 4), cross-level interaction effects were used to examine whether the associations between individual variables and social distance were moderated by context variables. Of all interaction effects in this model, only one interaction between psychiatric rehabilitation services density and perceived dangerousness in association with social distance was obtained. This means that individuals who had high perceived dangerousness and lived in areas with high density of psychiatric rehabilitation services may have high social distance.

## 4. Discussion

There is limited previous literature examining the influences of the regional context and individual characteristics on the social distance of depression. As far as we know, this is the first study to explore the influence of both of the individual-level and the contextual-level factors with social distancing from persons with depression using a multilevel approach. Consistent with previous studies [17,64], the desire for social distance was higher in men than in women. Women were found to be more empathetic than men towards persons with mental illness in terms of attitudes of open-mindedness and preparedness to integrate persons with mental illness in the community [16]. Women also had more positive attitudes towards patients with depression than men [65]. In addition, men may have less exposure to depression than women, which would result in limited experience and information about the condition [66] and increase their desire for social distance [63]. With regard to age, our results are consistent with those reported earlier [18,19,67,68], i.e., older adults express greater social distancing from patients with depression than younger adults. Older people may tend to be rigid in their belief that people with mental illness are aggressive and violent. Such beliefs may discourage them from approaching people with mental illness [69]. Additionally, their physical fragility may make them feel vulnerable to the potentially violent behaviors of patients with mental illness [69].

Second, our findings indicated that there was no significant correlation between educational attainment and social distance. This result is consistent with that of previous studies [47,70], while some other studies showed that the higher the educational level of people, the greater the social distancing from patients [71,72]. One possible explanation is that people with a low education level have different beliefs about mental illness from those with higher level of education. For example, a previous study showed that people with low level of education are likely to believe that a weak will is a possible cause of mental disorders such as depression or schizophrenia [73]. Another study showed that people with higher education hold more restrictive attitudes than illiterate people, so highly educated people do not trust patients with mental illness easily [74]. These potential beliefs about mental illness may be possible factors that mediate the relationship between education attainment and social distance. Another possible explanation for the lack of significance is that the educational attainment in the present sample was generally high school or above (*n* = 732, 91.50%), which may have affected the statistical accuracy of the bivariate correlation analysis.

The current study found that people with more perceived dangerousness and emotional reaction of fear were associated with greater social distancing from patients with depression, which is in line with previous studies in Western countries [22,23,26,28]. The perceived dangerousness towards patients with mental illness indicates the perception of disease severity, where many people have strong beliefs that mental illness is untreatable [75]. Such beliefs and attitudes may lead to increased social distancing from patients. In addition to perceived dangerousness, people with increased emotional reactions of fear were associated with increased social distance. People with fear mainly think of the violence caused by patients who are regarded as unsafe, and thus should be kept at a distance [76,77,78]. The research found that people diagnosed with depression were roughly three times more likely than the general population to commit violent crimes including robbery, sexual offenses and assault [79]. However, people often overestimate the risk, and most patients do not pose a threat to people [80]. Compared with previous studies in Western countries, a consistent pattern of these results was demonstrated in Chinese society. In recent years, research has indicated that the social distancing from patients with mental illness is different among various ethnicities and cultures [81]. With the rise of global internationalization, cross-cultural research to understand different ethnic beliefs on mental illness is important [82]. The association between perceived dangerousness and emotional reaction of fear and social distance in different ethnicities requires further studies.

Our findings indicated that the main effect of the density of psychiatric rehabilitation services on social distance was not significant. However, we found that there was a cross-level interaction between the density of psychiatric rehabilitation services and perceived dangerousness in the association with social distance. In this study, we focused on the factors influencing social distancing from patients with depression among the general public, although not all patients in psychiatric rehabilitation services may have depression. Recent research has pointed out that contact with one mental illness will not only affect the stigma of that mental illness, but also produce a transfer effect that would also influence the stigma towards other mental illnesses [83]. The association between perceived dangerousness and social distance was pronounced in the regions with high density of psychiatric rehabilitation services. Perceptions of dangerousness may be influenced by different sources of information. Dangerousness—which is associated with psychiatric symptoms or deviant behavior—is one of these sources [84]. Information about psychiatric service use (e.g., information that a person has received treatment at a psychiatric hospital) is another important source of perception of dangerousness [84,85]. People who believe in dangerousness avoid contacting patients with mental illness [86]. If the belief that people with mental illness are dangerous, then people who have more contact with people with mental illness should perceive them as more dangerous than those who have less contact [87]. In our study, people who lived in a county or city with high density of psychiatric rehabilitation services may have a high chance of contact with patients with mental illness. This kind of naturalistic contact without a carefully planned process of disclosure and education may increase the stigma of mental illness because these patients are actively symptomatic and cannot hide their illness in the way that someone who has recovered [88].

In our study, we found that the higher the degree of urbanization in the region, the higher the desire among the public for social distancing from patients with depression. Previous research illustrated that people living in urban areas are more unwilling to work with patients with mental illness than people living in rural areas [46]. Rural residents also show a supportive, tolerant attitude in assisting patients on the path of recovery [46]. Furthermore, rural living conditions with opportunities for manual and agricultural labor could provide perceived useful opportunities for persons affected by mental illness [89]. In contrast, a wide range of technology-based industries is conducive for urban growth, and this brings about a competitive job situation in urban areas [89,90]. We infer that people with mental illness in rural areas can be better integrated into the local society in rural areas than in high-pressure urban areas; hence, rural residents may show reduced social distancing from these people.

Several methodological limitations should be considered when drawing inferences from the findings of this study. First, this study has a cross-sectional design, making it impossible to draw definitive conclusions about the causal relationship between factors. More research is needed with longitudinal data to better understand the relative associations between individual-level and context-level factors and social distancing from patients with depression. Second, the limited numbers of the analyzed regions were the result of the data collection methodology. Small context-level sample sizes can lead to not only bias, but also convergence issues. Future studies with a larger number of regions are necessary to obtain robust results. Third, it is also possible that self-reports of stigma may be especially vulnerable to social desirability bias. The suggestion for future study is to explore more variables related to stigma and the contextual factors of residential area, such as social cohesion and media coverage of mental illness in the community. These factors may affect the public’s stigma attitude towards patients with depression. It is necessary to use different measurement tools to clarify the relevant influence mechanism of the individual-level and contextual-level variables on the stigma of mental illness.

## 5. Conclusions

This study highlights individual-level and contextual-level factors associated with social distance in the Chinese community. Our findings provide valuable information for future related research, and the effect of the interaction between individual-level and contextual-level factors on social distance should be taken into account. Our findings also provide evidence-based data for policies and interventions with the aim of reducing the public stigma of depression.

## Figures and Tables

**Table 1:** Characteristics of the participants (*n* = 800).

Variable	*n*	%		
Sex				
Male	400	50.00		
Female	400	50.00		
Age (years)				
20–29	152	19.00		
30–39	192	24.00		
40–49	181	22.63		
50–59	182	22.75		
60–65	93	11.63		
Educational attainment				
Primary education or below	14	1.75		
Secondary education	54	6.75		
High school	242	30.25		
University degree or above	490	61.25		
Variable	M	SD	Skewness	Kurtosis
Perceived dangerousness	6.00	1.53	0.52	−0.62
Emotional reaction of fear	2.62	1.09	0.55	−1.09
Social distance	21.06	4.30	0.56	0.28

**Table 2:** Bivariate correlation analysis of individual-level variables and social distance.

Variable	M	SD	r	*p* Value
Sex			−0.07 ^†^	0.04
Male	21.36	4.52		
Female	20.76	4.06		
Age (years)			0.23 ^‡^	<0.001
20–29	19.01	3.70		
30–39	20.97	4.19		
40–49	21.73	4.37		
50–59	21.36	3.95		
60–65	22.69	4.81		
Educational attainment			−0.03 ^‡^	0.36
Primary education or below	21.86	3.35		
Secondary education	20.94	4.44		
High school	21.27	4.32		
University degree or above	20.94	4.31		
Perceived dangerousness	–	–	0.22 ^§^	<0.001
Emotional reaction of fear	–	–	0.20 ^§^	<0.001

^†^ Point bi-serial correlation coefficient; ^‡^ Spearman’s rank correlation coefficient; ^§^ Pearson’s correlation coefficient.

**Table 3:** Multilevel analysis of individual-level and context-level variables on social distance among general public.

Variable (Ref.)	Model 1	Model 2	Model 3 ^†^	Model 3 ^‡^	Model 4 ^†^	Model 4 ^‡^
	*β (SE)*	*β (SE)*	*β (SE)*	*β (SE)*	*β (SE)*	*β (SE)*
Fixed effect						
Individual-level						
Sex (male)		−0.70 * (0.29)	−0.70 * (0.29)	−0.72 * (0.29)	−0.59 (0.66)	−1.00 (1.03)
Age		0.75 *** (0.11)	0.74 *** (0.11)	0.72 *** (0.11)	0.27 (0.26)	1.26 ** (0.42)
Perceived dangerousness		0.51 *** (0.09)	0.51 *** (0.09)	0.50 *** (0.09)	0.005 (0.22)	0.25 (0.34)
Emotional reaction of fear		0.67 *** (0.13)	0.67 *** (0.13)	0.68 *** (0.13)	0.97 ** (0.30)	0.84 (0.48)
Context-level						
The density of psychiatric rehabilitation services			0.20 (0.30)		−2.85 (1.58)	
The degree of urbanization				1.40 * (0.67)		1.18 (3.91)
Cross-level						
Sex × the density of psychiatric rehabilitation services					−0.08 (0.54)	
Age × the density of psychiatric rehabilitation services					0.38 (0.19)	
Perceived dangerousness × the density of psychiatric rehabilitation services					0.46 * (0.17)	
Emotional reaction of fear × the density of psychiatric rehabilitation services					−0.26 (0.24)	
Sex × the density of urbanization						0.41 (1.34)
Age × the density of urbanization						−0.70 (0.53)
Perceived dangerousness × the density of urbanization						0.36 (0.44)
Emotional reaction of fear × the density of urbanization						−0.22 (0.61)
Random effect						
Between-group variance	0.22 *	0.10 *	0.11 *	0.08 *	0.15 *	0.13 *
Within-group variance	18.31	16.10	16.10	16.12	15.92	16.15
Intraclass correlation (%)	1.19	0.62	0.68	0.49	0.93	0.80
Deviance	4603.87	4502.32	4502.43	4497.63	4494.55	4491.04

Note: Model 1 = null model; Model 2 = fixed slope regression model; Model 3 = intercept as outcomes regression model; Model 4 = model with nonrandomly varying slopes.; ^†^ psychiatric rehabilitation services density as context-level variable; ^‡^ urbanization degree as context-level variable.; * *p <* 0.05. ** *p <* 0.01. *** *p <* 0.001.

## Appendix A

**The case vignette:** Mei (or Daxiong) is 30 years old and is not married. S/he used to regularly help her/his father work on the store, but for the last 10–15 days, s/he has not been going to work. For the last 2–3 months, s/he was staying alone and aloof. S/he has not been bathing regularly and sometimes becomes aggressive for no apparent reason. S/he never used to behave in this way. On several occasions, her/his father has found her/him talking to her/himself when nobody else was around. S/he has become suspicious of others and says that people are talking about her/him. For the last one week s/he has refused to eat food as s/he suspects her/his food is being poisoned by the neighbors. Mei (or Daxiong) is 30 years old and was fine until six months ago when s/he began to feel tired all the time. S/he says that s/he is sad and has lost interest in life. Even her/his children and family do not make her/him feel happy. S/he cannot sleep and s/he has lost the taste for foods s/he used to love. S/he has also lost interest in cooking because s/he cannot concentrate. Sometimes s/he feels like jumping into the well to end her/his life.
